# Analysis of Xq27-28 linkage in the international consortium for prostate cancer genetics (ICPCG) families

**DOI:** 10.1186/1471-2350-13-46

**Published:** 2012-06-19

**Authors:** Joan E Bailey-Wilson, Erica J Childs, Cheryl D Cropp, Daniel J Schaid, Jianfeng Xu, Nicola J Camp, Lisa A Cannon-Albright, James M Farnham, Asha George, Isaac Powell, John D Carpten, Graham G Giles, John L Hopper, Gianluca Severi, Dallas R English, William D Foulkes, Lovise Mæhle, Pål Møller, Rosalind Eeles, Douglas Easton, Michelle Guy, Steve Edwards, Michael D Badzioch, Alice S Whittemore, Ingrid Oakley-Girvan, Chih-Lin Hsieh, Latchezar Dimitrov, Janet L Stanford, Danielle M Karyadi, Kerry Deutsch, Laura McIntosh, Elaine A Ostrander, Kathleen E Wiley, Sarah D Isaacs, Patrick C Walsh, Stephen N Thibodeau, Shannon K McDonnell, Scott Hebbring, Ethan M Lange, Kathleen A Cooney, Teuvo LJ Tammela, Johanna Schleutker, Christiane Maier, Sylvia Bochum, Josef Hoegel, Henrik Grönberg, Fredrik Wiklund, Monica Emanuelsson, Geraldine Cancel-Tassin, Antoine Valeri, Olivier Cussenot, William B Isaacs

**Affiliations:** 1 Inherited Disease Research Branch, National Human Genome Research Institute, National Institutes of Health, Baltimore, MD, 21224, USA; 2 Johns Hopkins Bloomberg School of Public Health, Baltimore, MD, USA; 3 Department of Health Sciences Research, Mayo Clinic, Rochester, MN, 55905, USA; 4 Data Coordinating Center for the ICPCG and Center for Human Genomics, Wake Forest University School of Medicine, Winston-Salem, NC, 27157, USA; 5 University of Utah ICPCG Group and Division of Genetic Epidemiology, University of Utah School of Medicine, Salt Lake City, UT, USA; 6 George E. Wahlen Department of Veterans Affairs Medical Center, Salt Lake City, UT, USA; 7 African American Hereditary Prostate Cancer ICPCG Group, Phoenix, AZ, USA; 8 Fox Chase Cancer Center, Philadelphia, PA, USA; 9 Karmanos Cancer Institute, Wayne State University, Detroit, MI, USA; 10 Translational Genomics Research Institute, Genetic Basis of Human Disease Research Division, Phoenix, AZ, USA; 11 ACTANE consortium; 12 Cancer Epidemiology Centre, Cancer Council Victoria, Melbourne, Australia; 13 Centre for Molecular, Environmental, Genetic and Analytic Epidemiology, School of Population Health, The University of Melbourne, Melbourne, Australia; 14 Program in Cancer Genetics, McGill University, Montreal, QC, Canada; 15 Department of Medical Genetics, Oslo University Hospital, The Norwegian Radium Hospital, Oslo,Norway; 16 Institute of Cancer Research and Royal Marsden NHS Foundation Trust, Surrey, UK; 17 Cancer Research UK Genetic Epidemiology Unit, Cambridge, UK; 18 Division of Medical Genetics, University of Washington Medical Center, Seattle, WA, USA; 19 BC/CA/HI ICPCG Group, Stanford, CA, USA; 20 Department of Health Research and Policy, Stanford School of Medicine, Stanford, CA, USA; 21 Stanford Cancer Institute, Stanford School of Medicine, Stanford, CA, USA; 22 Department of Urology and Department of Biochemistry and Molecular Biology, University of Southern California, Los Ageles, CA, USA; 23 FHCRC ICPCG Group, Seattle, WA, USA; 24 Fred Hutchinson Cancer Research Center, Division of Public Health Sciences, Seattle, WA, USA; 25 Cancer Genetics Branch, National Human Genome Research Institute, National Institutes of Health, Bethesda, MD, USA; 26 Institute for Systems Biology, Seattle, WA, USA; 27 Johns Hopkins University ICPCG Group and Department of Urology, Johns Hopkins Medical Institutions, Baltimore, MD, USA; 28 Mayo Clinic, Rochester, MN, USA; 29 University of Michigan ICPCG Group, Ann Arbor, MI, USA; 30 Department of Genetics, University of North Carolina, Chapel Hill, NC, USA; 31 University of Michigan, Ann Arbor, MI, USA; 32 University of Tampere ICPCG Group, Tampere, Finland; 33 Institute of Biomedical Technology, University of Tampere, Tampere, Finland; 34 Centre for Laboratory Medicine and Department of Urology, Tampere University Hospital, Tampere, Finland; 35 University of Ulm ICPCG Group, Ulm, Germany; 36 Dept of Urology, University of Ulm, Ulm, Germany; 37 Institute of Human Genetics, University of Ulm, Ulm, Germany; 38 Department of Medical Epidemiology and Biostatistics, Karolinska Institutet, Stockholm, Sweden; 39 Oncologic Centre, Umeå University, Umeå, Sweden; 40 CeRePP ICPCG Group, 75020, Paris, France; 41 Hopital Tenon, Assistance Publique-Hopitaux de Paris, 75020, Paris, France; 42 Cancer Prevention Institute of California

## Abstract

**Background:**

Genetic variants are likely to contribute to a portion of prostate cancer risk. Full elucidation of the genetic etiology of prostate cancer is difficult because of incomplete penetrance and genetic and phenotypic heterogeneity. Current evidence suggests that genetic linkage to prostate cancer has been found on several chromosomes including the X; however, identification of causative genes has been elusive.

**Methods:**

Parametric and non-parametric linkage analyses were performed using 26 microsatellite markers in each of 11 groups of multiple-case prostate cancer families from the International Consortium for Prostate Cancer Genetics (ICPCG). Meta-analyses of the resultant family-specific linkage statistics across the entire 1,323 families and in several predefined subsets were then performed.

**Results:**

Meta-analyses of linkage statistics resulted in a maximum parametric heterogeneity lod score (HLOD) of 1.28, and an allele-sharing lod score (LOD) of 2.0 in favor of linkage to Xq27-q28 at 138 cM. In subset analyses, families with average age at onset less than 65 years exhibited a maximum HLOD of 1.8 (at 138 cM) versus a maximum regional HLOD of only 0.32 in families with average age at onset of 65 years or older. Surprisingly, the subset of families with only 2–3 affected men and some evidence of male-to-male transmission of prostate cancer gave the strongest evidence of linkage to the region (HLOD = 3.24, 134 cM). For this subset, the HLOD was slightly increased (HLOD = 3.47 at 134 cM) when families used in the original published report of linkage to Xq27-28 were excluded.

**Conclusions:**

Although there was not strong support for linkage to the Xq27-28 region in the complete set of families, the subset of families with earlier age at onset exhibited more evidence of linkage than families with later onset of disease. A subset of families with 2–3 affected individuals and with some evidence of male to male disease transmission showed stronger linkage signals. Our results suggest that the genetic basis for prostate cancer in our families is much more complex than a single susceptibility locus on the X chromosome, and that future explorations of the Xq27-28 region should focus on the subset of families identified here with the strongest evidence of linkage to this region.

## Background

Prostate cancer (PC) is the most common male cancer in developed countries [1]. In the United States, each year there are over 200,000 newly diagnosed cases and over 30,000 deaths attributable to prostate cancer [2]. Family history, along with older age and African-American ancestry, are the most important risk factors established to date. Inherited genetic factors might account for a proportion of the familial risk, but it has been very difficult to discover the actual genetic basis of prostate cancer probably due to the large number of loci involved, the incomplete and possibly low penetrance associated with these loci, and the likely clinical and genetic heterogeneity of this disease.

In 1996, the first prostate cancer linkage report implicated chromosome 1q23-25 [3], but subsequent linkage studies have found contradictory conclusions. In this same year the International Consortium for Prostate Cancer Genetics (ICPCG), consisting of researchers from 11 groups around the world, was formed. With the initial aim of examining linkage and trying to replicate previous linkage findings the ICPCG pooled 1,323 pedigrees with clinically- (but not genetically-) defined “hereditary prostate cancer” (HPC). Given the large number of families in this dataset, it was hoped that this would provide increased power to confirm or exclude linkage, and to allow for informative linkage analyses of large homogeneous subsets in an attempt to control for some of the likely heterogeneity that would otherwise weaken the ability to detect linkage. The ICPCG analysis of 775 families supported the finding of a prostate cancer–susceptibility gene linked to 1q24-25 in a defined subset of prostate cancer families with early age at onset, at least 5 affected relatives and evidence of male-to-male transmission [4]. The *RNASEL* gene was later implicated as harboring rare variant alleles that increase risk of prostate cancer and may account for this linkage signal [5]. Evidence has been accumulating in support of *RNASEL* as a prostate cancer risk locus, with several recent large case–control and cohort studies and a very large meta-analysis all showing significant associations of prostate cancer risk with polymorphisms in this locus [6-10].

Several other susceptibility loci presumed to contain rare variants of large effect on individual risk of prostate cancer have been suggested [3,4,11-42] and reviewed elsewhere [30,41,42]. In addition, recent genome-wide association studies (GWAS) have implicated multiple loci at which there are common variants (single nucleotide polymorphisms; SNPs) that are not necessarily functional but are associated with small effects on individual risk of prostate cancer [43-49]. For a review see Varghese and Easton [50]. Work is proceeding to try to identify more susceptibility loci, by GWAS using common SNP risk alleles, by conventional linkage analyses aimed at detecting genes with rare, high-penetrance risk alleles and by whole exome and whole genome sequencing analyses that can be used in conjunction with linkage and GWAS results.

In 1998, a study of 360 multiple-case families found evidence for a prostate cancer susceptibility locus on chromosome X in the region Xq27-q28 (*HPCX*) [38]. A subset of 52 Finnish families from this study was used to examine whether phenotypic subsets of families exhibited different evidence for linkage to this region. This study showed that families with no male-to-male (NMM) transmission and late age of onset of prostate cancer (> 65 years) exhibited stronger evidence of linkage to the Xq27-28 region than did the complete set of families [33]. There have been five replication studies, four of which supported linkage of prostate cancer susceptibility to this region [6,21,51-53] with the study of large Utah pedigrees yielding independent genome-wide significant evidence of linkage [21], and one which did not support linkage to this region [12]. A fine-mapping study in the Finnish population examined association of prostate cancer to microsatellite markers in the *HPCX* Xq27-28 region using 108 independent prostate cancer patients selected from families with multiple affected men (55 were from the linkage study above) and 257 controls (anonymous, healthy male blood donors) from the same Finnish population. Significant association was observed for two markers in the region, DXS1205 (p = 0.0003) and bG82i1.1 (p = 0.0006), with stronger association observed at DXS1205 in the subset of 60 cases from families with no evidence of male-to-male transmission (p = 0.0002) [11]. Association of these two markers with prostate cancer risk has been replicated in an Ashkenazi Jewish founder population [6]. Positive associations were observed for allele 135 of the bG82i1.1 marker (OR = 1.77, P = 0.01) and allele 188 of DXS1205 (OR = 1.65, P = 0.02) in 979 prostate cancer cases and 1,251 controls.

Under the Xq27-q28 linkage peak is a region of ~750 kb containing five *SPANX* genes (*SPANX-A1, -A2, -B, -C*, and *-D*). The *SPANX* genes encode nucleus-associated sperm proteins and their expression has been detected in a variety of cancers. While they were originally suggested as candidate genes for the *HPCX* susceptibility locus [54], more recent work has found no association between prostate cancer and mutations in any of these genes [55]. However, a more complex involvement of these genes is possible. Putative candidate genes for association with prostate cancer have been found on other regions of the X chromosome.

Gudmundsson *et al*. conducted a genome-wide SNP association study of prostate cancer in over 23,000 Icelanders followed by a separate replication study. Of the two novel SNPs identified by this study, one, rs5945572, was found on XP11.22 (odds ratio (OR) = 1.23) [56]. Eeles *et al*. also found association to this region in a large GWAS [47]. However, the odds ratios for the risk genotypes at this putative locus are quite small and not likely to be responsible for the linkage signal observed on Xq in highly aggregated pedigrees.

The aims of this study were to examine the evidence for linkage of prostate cancer to chromosome X using 1,323 multiple-case prostate cancer families from the ICPCG and genotyping a consensus map of 25 microsatellite markers and using both parametric and non-parametric allele-sharing linkage analyses. The pedigree subsets evaluated were presence/absence of male-to-male disease transmission (a surrogate for X-linked inheritance), Carter criteria of HPC [57,58], average age at onset of affected men in the family (<65 years of age or ≥ 65 years), and number of men in a family with confirmed PC. Determining whether any of these subsets show stronger evidence of linkage to the region may guide the selection of cases for future mutational analysis in this region.

## Methods

This analysis was performed on 1,323 families with hereditary prostate cancer ascertained by 11 groups participating in the ICPCG. The process of ascertaining families and confirming diagnosis of prostate cancer differed among the groups, but in all samples, men were considered to be affected with prostate cancer only if medical records or death certificates could confirm the diagnosis. The 11 groups that participated in this linkage analysis are described elsewhere [59].

In the statistical analysis, all families were first analyzed together. In addition, several subsets of families were created based on pedigree characteristics. A pedigree was classified as satisfying the Carter criteria for hereditary prostate cancer [57,58] if at least one of the following conditions were met: 1) three consecutive generations of PC along a line of descent; 2) at least three first-degree relatives with a diagnosis of PC; 3) two or more relatives with a diagnosis of PC at age ≤ 55 years. Pedigrees were also classified according to whether transmission of PC in the family appeared consistent with X-linked transmission (yes versus no versus unclear). A pedigree was considered to be consistent with X-linked transmission if all affected males only had a family history of prostate cancer on the maternal side of the family so there was no evidence of male-to-male transmission of a prostate cancer risk allele. A pedigree was considered to be inconsistent with X-transmission if the family contained at least one affected father-affected son pair or if at least one affected son had an affected paternal uncle or paternal grandfather (male-to-male transmission). Pedigrees containing at least one male who had a family history of prostate cancer on both sides of his family (bilineal) were considered to be inconsistent with X-transmission. Pedigrees containing only a sibship of affected men with no information about the prostate cancer history on the maternal or paternal sides of the family were considered to be unclear for X-transmission. Pedigrees were also classified as to whether or not the average age at onset of affected men in the family was less than 65 years of age.

Each group had genotyped a different set of markers in the Xq27-q28 region. In order to use the available genotype data without re-genotyping a common panel of markers, a consensus map of the genetic markers from the different groups was created as follows. A total of 26 different markers on chromosome Xq (see Additional file 1: Table S1) were genotyped by ICPCG members. For our analysis, the order of these markers was determined from UCSC Goldenpath (version hg13, released Nov.14.2002). The marker distance was based on the deCode map [60]. All markers were successfully mapped to Goldenpath or deCode maps and cM distances were interpolated for some markers that were located on Goldenpath but not on the deCode map (Table 1). Because some groups did not have either the first or last markers from this consensus map, dummy non-informative markers (i.e., homozygous for all subjects) were used as anchors for these groups. This allowed us to align all group’s linkage files to the consensus map, allowing for different groups using different markers. All groups computed parametric multipoint LOD scores and non-parametric multipoint allele-sharing LOD scores at 1 cM intervals along the consensus map, using the GENEHUNTER-PLUS software [61-63] implemented in common PERL scripts. These analyses were repeated using only the 964 families that were not included in the original publication of linkage to the Xq27-28 region [38]. The output files containing pedigree-specific parametric LOD scores and intermediate files for computing nonparametric Kong and Cox allele sharing LOD’s for each pedigree were sent to the Data Coordinating Center, which then combined the data for the linkage analyses. The planned analyses were developed and approved by members of the ICPCG. 

**Table 1:** Markers used in the analysis with map location information (base pair locations from the UCSC Goldenpath version hg13, released Nov.14.2002 and cM locations from the deCode linkage map)

**Marker**	**bp Start**	**bp End**	**deCode cM Position**	**interpolated cM position**
**DXS1216**	66597577	66597945	82.98	82.98
**DXS6800**	76721582	76721893	86.84	86.84
**DXS986**	77422288	77422628	86.84	86.84
**DXS990**	91036325	91036548	94.92	94.92
**DXS6789**	93484970	93485285	96.95	96.95
**DXS1106**	100764898	100765284		101.56
**DXS6797**	105514006	105514365	104.57	104.57
**GATA172D05**	111199673	111199793	110.42	110.42
**DXS8055**	112690834	112691203		112.66
**DXS1001**	117811961	117812315	120.35	120.35
**GATA165B12**	118830243	118830577	122.11	122.11
**DXS1047**	127020308	127020598		131.44
DXS1192	136312813	136313015	142.03	142.03
DXS1232	137224883	137225141		144.25
**DXS984**	137576507	137576708	145.8	145.8
**GATA31E08**	138167161	138167460		147.38
DXS1205	138195175	138195518	147.46	147.46
**DXS1227**	138735290	138735546	150.37	150.37
**DXS6751**	138961389	138961667		151.02
**DXS6798**	139563750	139564153		152.76
DXS8106	140116944	140117293	154.35	154.35
**DXS7127**	140874035	140874578		156.62
**DXS6806**	141366644	141366944	158.09	158.09
**DXS8043**	141885707	141885926		159.62
MXMAFMA113ZF5	142386616	142387004		161.1
**DXS1200**	143602429	143602828	164.69	164.69
DXS297	143861118	143861312		165.05
DXS731	145029666	145029754		166.65
MXMAFM323YF1	145482291	145482634		167.28
**DXS8091**	145497860	145498194	167.3	167.3
**AFM136yb10 (MXMAFM136YB10)**	146107588	146107837		168.95
MXMAFMA107XF5	146244529	146244891		169.32
**DXS1193**	146275270	146275535	169.4	169.4
DXS1123	146381305	146381484		169.78
**DXS8069**	147408020	147408348	173.44	173.44
DXS8011	147637858	147638200		176
DXS8103	147886112	147886447	178.77	178.77
AFMa225xh9 (MXMAFMA225XH9)	148302283	148302628		179.88
MXMAFMA082XA5	148619715	148620129		180.74
**DXS1073**	151414197	151414518	188.22	188.22

The allele frequencies for each marker in each group were estimated by counting alleles across all families, ignoring genetic relationships. All groups ran analyses using the widely-spaced genome-wide screening (GWS) markers (shown in bold text in Additional file 1: Table S1 and Table 1). Since some groups had also genotyped fine-mapping (FM) markers after finding suggestive evidence for linkage, our primary analyses used the GWS markers in order to attempt to eliminate any biases due to different information content across datasets. We also performed secondary analyses that included both GWS and FM markers. Since individual groups had fine-mapped at different densities and therefore obtained different levels of information content in their families, the secondary analyses were quite variable across samples in the amount of information available from the FM markers. This variability in marker density across studies could result in bias and so the combined analyses using the GWS markers are considered more reliable. We created two marker maps, one for the GWS markers and one for the FM markers (Table 1 shows the merged map of the GWS and FM markers).

A parametric model for dominant X-linked inheritance was used: the “Smith” model [3], with 2 liability classes, adapted to affecteds-only, X-linkage and a sex-limited trait. Multipoint parametric and non-parametric analyses were performed using GENEHUNTER-PLUS. After combining the results, multipoint heterogeneity LOD scores (HLODs) [63] were computed using the LOD scores from all sites. For the nonparametric allele-sharing LODs, the Kong and Cox allele sharing statistics were computed using output files from GENEHUNTER-PLUS [62].

## Results

In the nonparametric analyses, the analysis of all families using the GWS marker set resulted in an allele-sharing LOD of 2.0 in favor of linkage to Xq27-q28 at 138 cM, which is well below the commonly accepted threshold for claiming statistically significant evidence for linkage. Non-parametric analyses using the fine mapping (FM) marker set always resulted in the same or lower allele-sharing LODs (e.g. 1.22 at 125 cM in the complete dataset). The subsets that resulted in higher allele-sharing LODs for the GWS markers were the 732 families with 2–3 affecteds (allele-sharing LOD = 2.56 at 134 cM), the 627 families where mean age at onset was <65 years (allele-sharing LOD = 2.34 at 138 cM), and the subset of 288 families with 2–3 affecteds that appeared to exhibit male-to-male transmission (allele-sharing LOD = 3.49 at 134 cM). The subsets of families that appeared to exhibit patterns of prostate cancer consistent with X-linked inheritance did not have high positive allele-sharing LODs (Table 2).

**Table 2:** Nonparametric, maximum multipoint allele-sharing LODs (location, number of families) for subsets of families

**Number of affected males in family**	**Family consistent with X-linkage**	**Family unclear**	**Family not consistent with X-linkage**
2-3	0.26 (136 cM, 58)	0.17 (127 cM, 386)	**3.49 (134 cM, 288)**
4-5	0.12 (134 cM, 37)	0.71 (83 cM, 93)	0.19 (109 cM, 308)
6 or more	0.59 (176 cM, 9)	0.74 (109 cM, 5)	1.70 (153 cM, 139)

**Figure 1:**
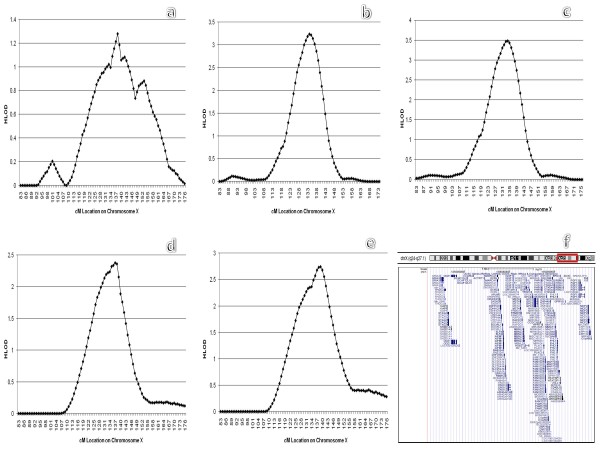
**Multipoint HLODs using the GWS marker set and the two-liability class parametric model: a**) using all families, **b**) in the subset of families with 2–3 affected males and possible male-to-male transmission of prostate cancer, **c**) in the subset of families that were not included in the original HPCX linkage paper [3,38] with 2–3 affected males and possible male-to-male transmission of prostate cancer, **d**) in the subset of families with 2–3 affected males that also meet the Carter criteria, **e**) in the subset of families with 2–3 affected males that also meet the Carter criteria and were not included in the original HPCX linkage paper [38]. Panel **f** is from the UCSC Genome Browser ( http://genome.ucsc.edu) on the Human February 2009 (GRCh37/hg19) Assembly of the human genome and shows the 2-LOD drop linkage interval from the HLOD graph in panel c. This region extends from approximately 122 cM to 144 cM, bounded by markers GATA165B12 and DXS1232, spanning base pair positions 120877968 to 139280361.

In the parametric, multipoint HLOD analyses, when all families were analyzed using the GWS marker set, the maximum HLOD was 1.28 at 138 cM (Figure 1a). When the FM marker set was used, the maximum HLOD was 0.45 at 125 cM in the complete set of families.

Subset analyses yielded larger HLOD scores in some subsets under this 2-liability class dominant parametric model. When using the GWS marker set, the subset of 104 families consistent with X-linked transmission, the 484 unclear families and the 735 non-X-linked (male-to-male transmission) families all gave positive HLODs, with a stronger signal observed in the latter group of families. When the FM marker set was used, the same pattern was observed: the subset of X-linkage transmission families gave a maximum HLOD = 0.246 at 132 cM, the unclear families gave a maximum HLOD = 0.142 at 143 cM, and the non-X-linkage families (male-to-male transmission families) yielded a maximum HLOD = 0.62 at 153 cM. The subset of 627 families with mean age at onset 65 years or younger gave HLOD = 1.8 at 138 cM using the GWS markers. The 696 families with mean age greater than 65 had maximum HLOD = 0.32 at 120 cM. Subdivisions based on the Carter criteria alone were not highly correlated with linkage evidence. Number of affected males in the family had a larger effect on linkage evidence, particularly when combined with pattern of transmission. The 732 families with 2–3 affecteds per family had maximum HLOD = 2.01 at 134 cM, whereas the 438 families with 4–5 affected males had HLOD = 0.1 at 168 cM and the 153 families with 6 or more affected males had HLOD = 1.4 at 153 cM. Consistent with the non-parametric analyses, the strongest evidence for linkage occurred in the subset of 288 families with 2–3 affected males and at least some evidence of male-to-male transmission: maximum multipoint HLOD = 3.24 at 134 cM (Figure 1b). Interestingly, when the analysis of this subset was restricted to the 248 families that were not included in the originally published linkage study [38], the maximum multipoint HLOD increased slightly to 3.47 at 134 cM, which exceeds the 3.3 value suggested by Lander and Kruglyak [64]for genome-wide significance (Figure 1c). The subset of 330 families with 2–3 affected males who also met the Carter criteria gave similarly strong linkage results with a maximum multipoint HLOD = 2.38 at 137 cM in all such families (Figure 1d) and 2.74 at 138 cM in the 284 families in this subset that were not included in the original X-linkage publication [38] (Figure 1e).

## Discussion

In the analyses presented here, there appeared to be little distinction between families with phenotypic segregation patterns consistent with X-linked inheritance (no male-to-male transmission) or those with evidence of male-to-male transmission when considering linkage evidence provided by those subsets of families for a PC susceptibility locus at Xq27-q28. Families with smaller numbers of affected men appeared to contribute the most evidence to linkage in this region. While classification of each family based on proportion of affected men out of total men old enough to be affected might provide more homogeneous subsets, this was not feasible for this study given the many sources of families with quite different ascertainment schemes and different degrees of completeness of pedigree data collection. In addition, given that prostate cancer is a late age at onset disease and is quite common, we are only able to assign “unaffected” status to men who are over the age of 75 years and who have a normal digital-rectal exam and normal PSA at that advanced age. There are small numbers of such well-characterized, elderly unaffected men in this set of families and most of the families do not have any of them, which would make a proportion misleading. Families with 2–3 affecteds per family had maximum HLOD = 2.01 at 134 cM. However, the strongest suggestion of linkage was observed in both the parametric and non-parametric analyses in families with 2–3 affecteds and possible male-to-male transmission. This pattern was observed whether we included the families from the initial linkage publication in the analysis or not. This subset of families, when excluding the families from the original linkage publication, had a multipoint HLOD of 3.47 at 134 cM. The HLOD in these new families was slightly over the Lander and Kruglyak threshold of 3.3 for genome-wide “significant” linkage but this threshold does not account for our multiple testing of different subsets, which likely requires a larger threshold to claim robust statistical evidence of linkage. However, this level of significance would meet the Lander and Kruglyak threshold for replication of a previously significant linkage (p = 0.01 or a LOD of approximately 1.0) even after correction for the multiple analyses. One candidate locus, *SPANXB1*, lies under this linkage peak. Since prostate cancer is fairly common, our analysis models allowed for the presence of sporadic cases in the families and the families with possible male-to-male transmission included some bilineal families. Thus, it is possible that in the male-to-male transmission family subsets, some families show sharing of X-chromosome markers among the maternally-related affected relative pairs in these pedigrees and no sharing among the paternally related affected pairs, thus giving evidence for X-linkage in these families. It appears that the evidence for linkage to Xq27-q28 is being driven mainly by families not included in the original linkage study [38] and these new families have had very few FM markers genotyped in this region (Additional file 1: Table S1). Interestingly, when the FM markers were added to the analyses of these same subsets, the HLODs no longer reached the Lander and Kruglyak genome-wide significance threshold. The information content when using only the GWS markers was fairly consistent across all families. However, when the FM markers were added, the information content differed greatly across groups of families and between the original and new families. An additional difference between the original and new families is that the families from the original linkage study had no markers genotyped more centromeric than 144 cM. Thus, it is possible that the differing position of linkage peaks between the analyses of the original and new families coupled with differential linkage information across these datasets is contributing to the inconsistent results.

**Figure 2:**
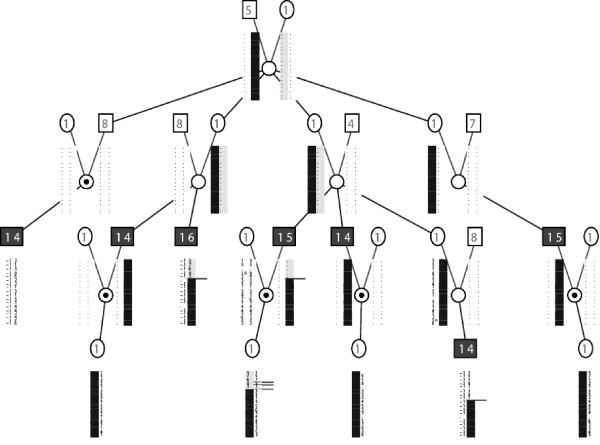
**Pedigree that exhibits both potential male-to-male transmission and potential maternal inheritance of prostate cancer.** In this family, with a maximum LOD of 1.7 in the HPCX region, all five maternally related affected males share a linked haplotype (shaded black) in this region and the one paternally-related affected male does not share this haplotype. The numbers in the shapes are liability classes based on affection status and age.

Our complete prostate cancer pedigree resource, with additional fine-mapping in the Xq27-28 region, provided, at best, modest suggestive evidence for linkage to this region. However, subsets of families with fewer affected individuals and paradoxically, families with some possible evidence of male-to-male disease transmission showed stronger linkage signals. This same result was observed in the analysis of very large Utah pedigrees [21], in which the best evidence for linkage was observed in the set of pedigrees with a maximum of 5 generations and an average of 2.5 genotyped prostate cancer cases. Although our finding of somewhat stronger linkage evidence in families with male-to-male disease transmission might not be sensible when considering a single locus on the X chromosome causing disease, this might indicate a more complex interaction between other susceptibility loci situated on the autosomes and a locus at Xq27-28. However, this finding might simply be due to high locus heterogeneity and/or important environmental risk factors in causation of prostate cancer, such that families segregating an X-linked risk allele may have at least one affected family member who is not a carrier of this risk allele and who is paternally related to another affected family member. Figure 2 shows one such pedigree that exhibits both potential male-to-male transmission and potential maternal inheritance of PC. In this family, with a maximum LOD of 1.7 in the *HPCX* region, all maternally related affected males share a linked haplotype in this region and the one paternally-related affected male does not share this haplotype. Finally, it is possible that the true causal allele in this region lies within the pseudoautosomal regions PAR2, which is near this linkage peak. Since female carriers cannot become affected with prostate cancer and since no marker loci have been genotyped in the PAR2 region in our families, our current data are inadequate for resolving this. 

## Conclusions

Although our results do not provide strong evidence for a major prostate cancer susceptibility gene located in this region of Xq27-28, there is some evidence for a locus that may contribute to risk in families with 2–3 affecteds and in some larger families such as the one in Figure 2. This locus does not appear linked to prostate cancer risk in a high proportion of larger HPC families with many affected males. Given these observations, gene identification efforts in highly penetrant families would be better targeted to other chromosomal regions, perhaps using whole-exome or whole genome DNA sequencing techniques. Gene identification at Xq27-28 should be aimed at the families with smaller numbers of affected men identified here as belonging to the most strongly linked subset and to specific large families with strongly positive LOD scores.

## Competing interests

The authors declare that they have no competing interests.

## Authors’ contributions

JEB-W, ELC, DJS, RE, DE, DJS, JLS, EAO, DK, LM, GGG, JLH, GS, HG, FW, ME, OC, GC-T, KAC, NJC, LAC-A, ASW, WBI and JX contributed to the design of the study. JEB-W, ELC, DJS and JX contributed to the performance of the meta-analyses. JEB-W, ELC, and CDC wrote the manuscript. DJS, SJM, RE, DE, JLS, EAO, GGG, WDF, JLH, GS, JS, TLT, GC-T, KAC, NJC, LAC-A, ASW, IO-G and WBI contributed to critical revision of the manuscript. MG, SE, JLS, EAO, DK, LM, GGG, WDF, JLH, GS, JS, TLT, OC, LM, PM, AV, KAC, LAC-A, KEW, SDI, PCW and WBI collected and maintained samples and data. EAO, DMK, SNT, SH, SB, GC-T, AV, KAC and CH performed or oversaw laboratory-based studies of samples. JLS, EAO, DMK, LM, SKM, CM, JH, HG, FW, GC-T, EML, NJC, JF, ASW, IO-G and MDB contributed to the linkage analyses at an ICPCG site. KD built and maintains a genotyping database at an ICPCG site. JX and LD coordinated and stored the linkage results from the contributing sites and harmonized the data. All authors have read and approved the final manuscript.

## Authors’ information

The members of the International Consortium for Prostate Cancer Genetics are as follows:

ACTANE Group: + Principal Investigators

UK, *Sutton*: S. Bullock, Q. Hope, S. Edwards, S. Bryant, S. Mulholland, S. Jugurnauth, N. Garcia, M. Guy, L. O'Brien, B. Gehr-Swain, A. Hall, R. Wilkinson, A. Ardern-Jones, D. Dearnaley, The UKGPCS Collaborators, British Association of Urological Surgeons' Section of Oncology, R. Eeles ^+^

UK, C*ambridge*: Chris Evans, M. Dawn Teare, Doug Easton + (Cancer Research UK Genetic Epidemiology Unit, Strangeways Research Labs, Cambridge)

Australia: John Hopper+, Graham Giles+, Dallas English, Gianluca Severi

(The Cancer Council of Victoria and The University of Melbourne, Carlton, Australia)

Canada: William D. Foulkes+, Nancy Hamel, Steven Narod, Jaques Simard+

(Department of Medical Genetics, Research Institute of the McGill University Health Centre, Montreal, Quebec; Women's College Hospital Research Institute, University of Toronto; Laboratoire de gÃ©nomique des cancers, Centre de Recherche du CHUQ, Laval University, Quebec City)

Texas: Mike Badzioch+, Chris Amos (MD Anderson Cancer Centre, Houston, TX and Division of Medical Genetics, University of Washington Medical Centre, Seattle, WA)

Norway, Oslo*:* Ketil Heimdal, Lovise Mæhle, Pål Møller + (Unit of Medical Genetics, Norwegian Radium Hospital, Oslo)

Norway, Ullevaal*:* Nicolai Wessel, Tone Andersen + (Dept of Oncology, Ullevaal University Hospital, Oslo)

EU Biomed: Tim Bishop+, The EU Biomed Prostate Cancer Linkage Consortium

(Cancer Research UKGenetic Epidemiology Laboratory, St James’ University Hospital, Leeds, UK)

BC/CA/HI Group: Raymond N. Balise^1^, Richard Gallagher^2^, Jerry Halpern^1^, Chih-lin Hsieh^3^, Laurence Kolonel^4^, Ingrid Oakley^5^, Dee West^1,5^, Alice S. Whittemore^1^ and Anna Wu^3^ (^1^Stanford University School of Medicine, Stanford, CA; ^2^British Columbia Cancer Center, Vancouver; ^3^University of Southern California, Los Angeles, CA; ^4^University of Hawaii, Honolulu, HI; ^5^Northern California Cancer Center, Union City, CA, Stanford, CA;)

CeRePP Group: Géraldine Cancel-Tassin, Antoine Valéri, Philippe Mangin, Olivier Cussenot (Centre de Recherche pour les Pathologies Prostatiques, Paris, France)

JHU Group: Kathleen E. Wiley, Sarah D. Isaacs, Marta Gielzak, Charles M. Ewing, Patrick C. Walsh, William B. Isaacs (Johns Hopkins Medical Institutions, Baltimore, MD)

Mayo Group: Daniel J. Schaid, Shannon K. McDonnell, Gerald B. Christensen, Julie M. Cunningham, Scott Hebbring, Jennifer C. Guenther, Stephen N. Thibodeau (Mayo Clinic, Rochester, MN)

Michigan Group: Ethan M. Lange^1^, Cralen C. Davis^1^, W. Mark Brown^1^, Cathryn H. Bock^2^, Kathleen A. Cooney^2^ (^1^Wake Forest University, Winston-Salem, NC; ^2^University of Michigan, Ann Arbor, MI)

Fred Hutchinson Cancer Research Center Group (PROGRESS): Kerry Deutsch^1^, Danielle M. Friedrichsen^2^, Suzanne Kolb^3^, Elaine A. Ostrander^2^, Lee Hood^1^, Janet L. Stanford^3^ (^1^Institute for Systems Biology, Seattle, WA; ^2^National Human Genome Research Institute, NIH, Bethesda, MD; ^3^Division of Public Health Sciences, Fred Hutchinson Cancer Research Center, Seattle, WA)

Tampere Group:Tiina Wahlfors^1^, Henna Mattila^1^, Virpi Laitinen^1^, Riikka Nurminen^1^, Daniel Fischer^1^, Teuvo L.J. Tammela^1^, Asha George^2^, Joan Bailey-Wilson^3^, Johanna Schleutker^1^ (^1^University of Tampere and Tampere University Hospital, Tampere, Finland; ^2^Fox Chase Cancer Center, Division of Population Science, Philadelphia, PA; ^3^Inherited Disease Research Branch, National Human Genome Research Institute, National Institutes of Health, Baltimore, MD)

Ulm Group: Ulm Group: Sylvia Bochum^1^, Thomas Paiss^2^, Josef Hoegel^1^, Florian Kurtz^1,3^, Manuel Luedeke^1,2^, Antje Rinckleb^1,2^, Kathleen Herkommer^2,3^, Walther Vogel^1^, Mark Schrader^2^, Christiane Maier^1,2^ (^1^Institut fuer Humangenetik, Universitätsklinikum Ulm, Ulm, Germany ^2^Urologische Klinik, Universitaetsklinik Ulm, Ulm, Germany, ^3^Urologische Klinik rechts der Isar, Technische Universitaet Muenchen, Munich, Germany)

Umeå/Karolinska Group: Fredrik Wiklund, Anders Bergh, Monica Emanuelsson, Ingela Göransson, Björn-Anders Jonsson, Fredrik Lindmark, Elisabeth Stenman, Henrik Grönberg(Umeå University, Umeå, Sweden and Karolinska Institutet, Stockholm, Sweden)

Utah Group: Lisa A. Cannon-Albright, Nicola J. Camp, James M. Farnham (University of Utah, Salt Lake City, UT)

Data Coordinating Center: Jianfeng Xu, Deborah A. Meyers, Bao-Li Chang, Aubrey R. Turner, Latchezar Dimitrov, Tamara S. Adams (Center for Human Genomics, Wake Forest University School of Medicine, Winston-Salem, NC)

Daniella Seminara (National Cancer Institute, Division of Cancer Control and Population Sciences, Bethesda, Maryland)

## Pre-publication history

The pre-publication history for this paper can be accessed here:

http://www.biomedcentral.com/1471-2350/13/46/prepub

## Supplementary Material

Additional file 1 **Table S1.** Markers genotyped and used in the linkage analyses by each data collection group [3]. Click here for file
