# Supplementary material for: Analysis of Xq27-28 linkage in the international consortium for prostate cancer genetics (ICPCG) families
Source: BMC Med Genet. 2012 Jun 19;13:46. doi: 10.1186/1471-2350-13-46 (PMC3495053; doi:10.1186/1471-2350-13-46)
Supplement: Additional file 1 — Table S1. Markers genotyped and used in the linkage analyses by each data collection group [3]. [file 1471-2350-13-46-S1.pdf]

Table 1 - Markers genotyped and used in the linkage analyses by each data collection group.

Marker	JHU 1998	Mayo 1998	Finn 1998	Swed 1998	BC/CA/HI	FHCRC	Finn	France	JHU (GW)	Mayo (GW)	Michigan	Sweden	UK	Ulm	Utah
**DXS1216***							**10**		**191**		**174**	**67**			
**DXS6800**						**186**							**64**		
**DXS986**							**10**		**191**	**161**	**174**	**67**			
**DXS990**							**10**		**191**	**161**	**174**	**67**			
**DXS6789**						**186**							**64**		
**DXS1106**							**10**		**191**	**161**	**174**	**67**			
**DXS6797**						**186**							**64**		
**GATA172D05**						**186**							**64**		
**DXS8055**							**10**		**191**	**161**	**174**	**67**			
**DXS1001**							**10**		**191**	**161**	**174**	**67**			
**GATA165B12**						**186**							**64**		
**DXS1047**						**186**	**10**		**191**	**161**	**174**	**67**	**64**		
DXS1192															146
DXS1232	139	123	57							161					
**DXS984**	**139**	**123**	**57**	**41**		**186**	**10**	**66**	**191**	**161**	**174**	**67**		**104**	**146**
**GATA31E08**					**100**	186							**64**		
DXS1205	139	123	57							161	153				
**DXS1227**							**10**		**191**	**161**	**174**	**67**			
**DXS6751**	**139**	**123**	**57**		**100**					161				**104**	
**DXS6798**	139		57												**146**
DXS8106	139	123	57		100	186		**66**		161				103	
**DXS7127**					**100**								**64**		
**DXS6806**	139	123	57	41		**186**				161					
**DXS8043**	**139**	**123**	**57**	**41**			**10**		**191**	**161**	**174**	**67**			
MXMAF MA113ZF5			57							161					
**DXS1200**	**139**	**123**	**57**	**41**	**100**	**186**		**66**		**161**	**153**			**104**	**146**
DXS297	139	123	57			186				161					
DXS731										161					

MXMAFM323YF1										161					
**DXS8091**	**139**	**123**	**57**				**10**		**191**	**161**	**174**	**67**			
**AFM136yb10 (MXMAFM136YB10)**	139	123	57							161					**146**
MXMAFMA107XF5										161					
**DXS1193**	139		57	41	**100**	**186**					153			**103**	146
DXS1123										161					
**DXS8069**	139	123	57	41		186				161					146
DXS8011	139		57												
DXS8103	**139**	**123**	**57**	**41**		**186**				**161**					**146**
AFMa225xh9 (MXMAFMA225XH9)	139	123	57							161					146
MXMAFMA082XA5										161					
**DXS1073**							**10**		**191**	**161**	**174**	**67**			**146**

* Yellow-shaded, bolded text indicates markers included in the GWS marker set for each sample and blue-shaded, non-bolded text indicates markers also included in the GWS plus fine-mapping marker set. Yellow-shaded, bolded text of the marker name indicates that it served as a GWS marker for at least one sample, but some of these markers served only as finemapping markers in some densely typed data sets. The first four columns indicate families that were included in the original linkage paper [3]. Numbers in the table represent the number of families genotyped for each marker in each dataset.
